# Editorial: Immunotherapy and small molecule inhibitors as combinational cancer therapeutics

**DOI:** 10.3389/fimmu.2023.1270182

**Published:** 2023-08-22

**Authors:** Mohd Wajid Ali Khan, Mohammad Azhar Aziz, Saravanan Rajendrasozhan

**Affiliations:** ^1^ Department of Chemistry, College of Science, University of Ha’il, Ha’il, Saudi Arabia; ^2^ Medical and Diagnostic Research Centre, University of Ha’il, Ha’il, Saudi Arabia; ^3^ Interdisciplinary Nanotechnology Center, Zakir Husain College of Engineering and Technology, Aligarh Muslim University, Aligarh, India

**Keywords:** immunotherapy, cancer therapeutics, immune check point, small molecules, PD1 (programmed cell death protein 1)

Cancer is a multidimensional chronic disease, with various factors contributing to its progression. The immune system plays a crucial role in identifying and eliminating tumor cells. However, aggressive cancers and advanced stages can pose challenges in treatment due to poor cancer cell targeting. To overcome this, research focus has shifted to developing novel small molecules which can further enhance immune system function, increasing the efficacy of cancer immunotherapies, and offering promising therapeutic options for different cancer types.

Cancer immunotherapy based on checkpoint inhibitors, aims to restore the normal defense of the body, to counter cancer cells. Programmed cell death protein 1/programmed death–ligand 1 (PD1/PDL1) check point inhibitors are already in clinical use. There are ongoing efforts to identify several other checkpoints that tumor cells exploit to mask and restrict the immune response. One of these checkpoints reviewed in this Research Topic is the CD200/CD200R pathway (Choe et al.). Authors introduced and reviewed the mechanism of CD200 checkpoint inhibition by tumor cells. Targeting of CD200/CD200R pathway would lead to antitumor activity within its environment. In another paper, evidence is provided to support the use of the drug Afatinib as a combination therapy in hepatocellular carcinoma along with PD1 inhibitors (Yu et al.). Another case report demonstrated the beneficial use of PD1 inhibitor along with two other drugs in a patient with BRAF and NRAS mutations (Gui et al.).

Cancer metabolism has been explored as a precision based therapeutic target. In this Research Topic, the role of Afatinib has been explained in terms of its link in D-Glutamine and D-Glutamic acid metabolism. Separately, another study took up the task to delineate the role of ENO1 in pancreatic cancer (PC) (Song et al.). The α-enolase (ENO1) is one of the key enzymes in the glycolytic pathway used by cancer cells for energy generation (Warburg effect). Overexpression of this gene has been associated with increased tumorigenesis in PC. The indispensable role of ENO1 in PC was proved by employing the knockout strategy. Thus, it can serve as a potential drug target to shut off glycolysis in cancer cells. However, the plasticity of cancer cells remains a concern and must be addressed when employing such approaches.

Abnormal glutamine metabolism has also been identified as a key factor in bladder cancer progression. The necessity to assess the prognosis and therapeutic efficacy of bladder cancer treatments based on an analysis of glutamine metabolism related genes is vital. The Cancer Genome Atlas was used to identify glutamine metabolism-related genes as prognostic markers, and established a novel Glutamine Metabolism Immunity Index (GMII) based on univariate and multivariate COX regression analyses. Candidate small-molecule drugs targeting the GMII core target proteins were identified based on molecular docking analysis. The GMII consisting of eight independent prognostic genes was established using molecular docking analysis as an excellent discriminating tool for predicting the survival of patients with bladder cancer. These results showed 12 potential small-molecule drugs that could bind to three of the GMII core target proteins (Xu et al.).

Cyclooxygenases-2 (COX-2) and Prostaglandin E2 (PGE2), which are important in chronic inflammatory diseases, can increase tumor incidence and promote tumor growth and metastasis. The COX-2-PGE2 pathway promotes tumor immune evasion by regulating myeloid-derived suppressor cells, lymphocytes (CD8+ T cells, CD4+ T cells and natural killer cells), and antigen presenting cells (macrophages and dendritic cells). Based on conventional treatment, the addition of COX-2 inhibitors or EP antagonists may boost immunotherapy response in anti-tumor immune escape scenarios (Jin et al.).

Drug repurposing is one of the ideas that is underexplored in cancer therapy. With the availability of computational infrastructure and artificial intelligence based algorithms, it has never been so feasible to test various drugs and their combinations in different types and stages of cancer. The key to personalized medicine also lies in our ability to identify the right target in the right patient. The most challenging aspect of the targeted delivery of anti-cancer agents is achieving selective recognition of cancer cells. Recently, homotypic binding and specific protein-receptor interactions have emerged as the preferred method for delivering anti-cancer drugs. To effectively use cancer cell membrane encapsulated nanoparticles (CCMEN) as a delivery strategy, accurately predicting their selective targeting efficiency is of utmost importance. Eleven high-priority glioblastoma cell surface antigens were selected for probabilistic modeling based on relevance, expression levels availability, and crystal structure information from literature and databases. A new term, Breakeven point (BEP), was introduced as a characteristic of typical cancer cell membrane encapsulated delivery agents. The model’s predictions closely matched the experimentally observed values within a range of ±7% for both experimental test culture types. The probabilistic model efficiently predicts the directional preference of nanoparticle-coated cancer cell membranes (GCC membrane) for glioblastoma. It can be easily adapted for other cancer types involving CCMEN as delivery agents for potential immunotherapy, offering greater specificity in future cancer treatment development (Khan et al.).

Tumors with limited immune cell infiltration and immune activity may be due to restricting anti-CD40 agonistic antibody (αCD40) immune activation by releasing certain tumor antigens. Dendritic cells (DC) can be activated through αCD40 and stimulate antigen presentation, concomitantly activating cytotoxic CD8 cells. Authors found that activation of β-2 adrenergic receptor (β2AR) initiates the signaling of CD40 in DCs through direct inhibition of IkBa phosphorylation and indirect upregulating of phosphorylated-cAMP response element binding protein (pCREB). The alteration in the CD40 pathways was observed when a pan β-Blocker ‘propranolol’, was introduced, causing better tumor regression, enhanced influx of effector T cells, as well as decreasing the number of regulatory T cells as compared to monotherapy (Singh et al.).

Several studies focused on novel cancer immunotherapies to boost anti-tumor immunity, including adoptive cell-based therapies. Various immune cells and molecules are targeted to enhance tumor-specific T cell responses. Precision and personalized therapeutic approaches are a promising route for further innovation in immuno-oncology needed for treatment of aggressive cancers. Combinational therapies show promise, but extensive pre-clinical evaluation and testing is vital before clinical development and adoption.

## Author contributions

MK: Conceptualization, Supervision, Writing – original draft, Writing – review & editing. MA: Conceptualization, Writing – original draft, Writing – review & editing. SR: Writing – review & editing.

